# Anti-Human Leukocyte Antigen Antibody Detection from Terasaki’s Humoral Theory to Delisting Strategies in 2024

**DOI:** 10.3390/ijms26020630

**Published:** 2025-01-13

**Authors:** David San Segundo, Alejandra Comins-Boo, Marcos López-Hoyos

**Affiliations:** 1 Immunology Department, University Hospital Marqués de Valdecilla, 39008 Santander, Spain; david.sansegundo@scsalud.es (D.S.S.); alejandra.comins@scsalud.es (A.C.-B.); 2 Institute for Research Marqués de Valdecilla (IDIVAL), 39011 Santander, Spain; 3 Departamento de Biología Molecular, Universidad de Cantabria, 39011 Santander, Spain

**Keywords:** anti-HLA antibodies, donor-specific antibodies, Luminex, molecular mismatch, delisting

## Abstract

The human leukocyte antigen (HLA) system plays a critical role in transplant immunology, influencing outcomes through various immune-mediated rejection mechanisms. Hyperacute rejection is driven by preformed donor-specific antibodies (DSAs) targeting HLAs, leading to complement activation and graft loss within hours to days. Acute rejection typically occurs within six months post-transplantation, involving cellular and humoral responses, including the formation of de novo DSAs. Chronic rejection, a key factor in long-term graft failure, often involves class II DSAs and complex interactions between the innate and adaptive immune systems. Advancements in HLA antibody detection, particularly single antigen bead (SAB) assays, have improved the sensitivity and characterization of DSAs. However, these assays face challenges like false positives from denatured antigens and false negatives due to low antibody titers or complement competition. Furthermore, molecular mismatch (MM) analysis has emerged as a potential tool for refining donor–recipient compatibility but faces some issues such as a lack of standardization. Highly sensitized patients with calculated panel-reactive antibodies (cPRA) of 100% face barriers to transplantation. Strategies like serum dilution, novel therapies (e.g., Imlifidase), and delisting approaches could refine immunological risk assessment and delisting strategies are essential to expand transplant opportunities for these patients.

## 1. Introduction

### Alloresponse

**Figure 1:**
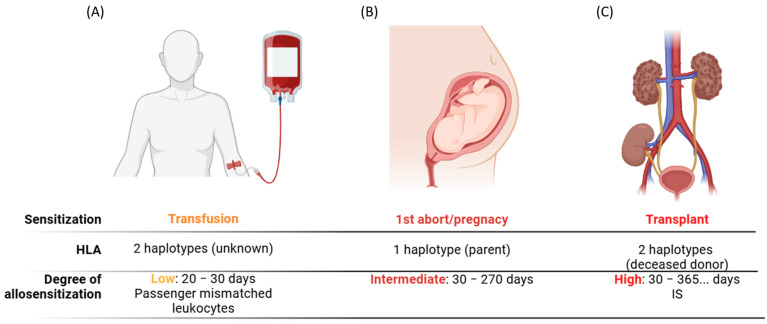
Mechanisms of allosensitization. Different ways of HLA allosensitization have been described. The differences in allosensitizations are based on different features: HLA mismatch load and time of sensitization. In transfusions, the HLA mismatch load could be up to 2 mismatched haplotypes, and the time of allosensitization stimulation is the lifespan of passenger-mismatched leukocytes (**A**). In pregnancy/aborts the HLA mismatch load is up to 1 mismatched haplotype, whereas the time of allosensitization ranges between 30 and 270 days (**B**). Finally, the allotransplant could have up to 2 mismatched haplotypes and the time of allosensitization is variable and controlled by immunosuppression (IS) in panel (**C**).

The main targets of the alloimmune response are the human leukocyte antigens (HLAs) [1]. The alloresponse triggered by HLA mismatches (MMs) represents the principal mechanism of allosensitization. Different types of allosensitization have been described, including previous transplants, pregnancy/miscarriages [2], transfusions [3], and some viral infections that mimic HLAs [4] (Figure 1). However, the duration and load of HLA MMs could determine the strength of the antibody response [5]. HLA mismatches can be recognized by the recipient’s immune system through two primary mechanisms. The first is direct allorecognition, where recipient T lymphocytes directly recognize donor HLA molecules. The second is indirect allorecognition, in which donor HLA molecules are processed by the recipient’s antigen-presenting cells and are presented as allo-peptides to recipient lymphocytes within the context of Major Histocompatibility complex (MHC) class II molecules [6]. HLA molecules play a critical role in antigen presentation within the immune system, enabling the initiation of an effector immune response.

HLA class I molecules (present in practically all nucleated cells) and HLA class II, expressed on antigen-presenting cells (APCs), can also be expressed by other cell subtypes upon activation, such as endothelial cells [7] and recently activated T cells [8]. This indicates that, although acute rejection generally occurs due to preformed antibodies acting against HLA class I [9], cases of early antibody-mediated rejection involving anti-HLA class II antibodies have also been described [10].

Following HLA MM exposure, professional APCs, such as dendritic cells (DCs), macrophages, and B cells, can recognize HLA MM and engulf, process, and drive peptide degradation to further present determinant epitopes of donor HLA MMs to specific CD4 lymphocytes with MHC-II restriction [11]. This interaction between DCs and specific-CD4 T cells would lead to CD4 activation and proliferation, and orchestrate a primary alloresponse. After the interaction between specific B and T cells, subsequent germinal center formation in regional lymph nodes is addressed. This process of priming specific naïve B cells lead to the activation and induction of anti-HLA antibody development. After the recall of the HLA MM antigen, a secondary response is mounted and the B cells undergo somatic hypermutation to fine-tune anti-HLA antibody specificity, potentially redirecting recognition to a different epitope of the HLA MM. The B cell class switch might create IgG1 and IgG3 anti-HLA-specific antibodies with a high ability to fix the complement. The feature of fixing the complement confers the antibodies with the possibility to have specific effector functions inducing lysis in those cells with the targeted antigen after membrane attack complex (MAC) formation. In addition to these potent complement-mediated activities, other IgG subclasses, such as IgG2 and IgG4, which exhibit reduced or absent complement-binding capabilities, retain other effector functions shared among IgG antibodies.

The persistence of HLA MM can trigger subsequent recall responses, including tertiary and quaternary responses. During these responses, B cells undergo additional rounds of somatic hypermutation and class switching, potentially targeting new epitopes on the HLA MM. The new B clones produce refined antibodies targeting HLA MM, and encounter a specific follicular helper CD4 T cells in the germinal center. These interactions may lead to the production of new anti-HLA antibodies, which can undergo isotype switching to non-complement-binding antibody subclasses [12]. The broad reactivity of the humoral response is due to the high degree of polymorphism and the significant similarity between different HLA molecules. This is a hurdle for those patients with expected retransplantation during their lifetime, such as children with end-stage renal disease. In such cases, minimizing the HLA MM load in the first allograft is recommended [13].

## 2. Allograft Rejection

Different types of allograft rejections have been established based on the timing after transplantation and the components of the immune response involved.

### 2.1. Hyperacute Rejection

Hyperacute rejection is primarily driven by the presence of preformed donor-specific antibodies in the recipient. The development of these antibodies could arise through natural processes, such as a reaction against ABO blood group antigens or after encountering foreign HLA molecules from a previous transplant, transfusion, or gestation [14] (Figure 1). Additionally, exposure to environmental antigens that exhibit molecular mimicry with HLA molecules has been implicated in antibody development [15]. This type of rejection occurs within hours to a few days after transplantation where preformed antibodies bind to endothelial cells or the transplanted tissue, leading to complement deposition MAC formation and cellular lysis. Furthermore, endothelial cell activation contributes to early graft thrombosis, exacerbating tissue damage and compromising graft function [16].

### 2.2. Acute Rejection

Acute rejection could be invoked within the first 6 months after transplantation. Following an early ischemia–reperfusion injury or innate immunity-mediated damage to endothelial cells, mononuclear cells infiltrate the interstitium, cross the basement membrane, and induce tubular damage [17]. At this point, mononuclear cells release soluble factors including chemoattractants which recruit neutrophils and cells from the adaptive immune system. In addition, natural killer (NK) cells can recognize allogeneic endothelial cells through the missing self pathway. This mechanism involves Killer Immunoglobulin-like Receptors (KIRs) binding to self or matched HLA molecules [18]. Finally, another type of allorecognition by NK cells and other innate immune cells is related to their Fc receptors (FcR), which could directly recognize alloantigen by both fixing and non-fixing donor-specific anti-HLA antibodies (DSA) [19,20].

In the absence of immunosuppression, cellular immunity could be activated, leading to allograft rejection within days [21]. Alloreactive T cells recognize donor MHC molecules either directly (via the direct pathway) or through processed donor MHC peptides presented by host antigen-presenting cells (APCs) through the indirect pathway. The frequency of alloreactive T cells is estimated to range from 1 to 10% [22], mainly due to the restricted thymus selection of T cells. After allorecognition, immune cells migrate to regional lymph nodes, where alloreactive B cells are activated. This activation induces the formation of germinal centers with de novo DSA production.

### 2.3. Chronic Rejection

Graft loss is related to chronic rejection, which can occur one year after transplantation. The underlying immunological mechanisms are complex and involve interactions between components of the innate and acquired immune systems [23]. Some animal models have demonstrated the role of NK cells in chronic antibody-mediated rejection [24], while different macrophage subsets have also been implicated in the progression of chronic rejection [25,26]. Additionally, the development of de novo class II DSA has been associated with chronic allograft rejection [27,28,29].

In summary, the humoral response could be involved in different types of allograft rejection in which the determining factor is the development of anti-HLA antibodies [30]. Hyperacute rejection, driven by preformed DSA, can be differentiated from acute rejection, which typically occurs in the early post-transplant period, often following the de novo development of class I DSA. In contrast, chronic rejection is generally associated with anti-HLA antibodies targeting class II molecules.

## 3. Anti-HLA Antibody Detection

The first assay developed for the detection of anti-HLA antibodies was complement-dependent cytotoxicity (CDC), in which complement-fixing anti-HLA antibodies are detected. A positive CDC crossmatch result indicates an increased risk of hyperacute rejection with a specificity and sensitivity of 98% and 75%, respectively [31]. The CDC detects IgG1, IgG3, and IgM anti-HLA antibodies that bind to donor HLA lymphocytes and elicit MAC formation and cellular lysis. However, IgM anti-HLA antibodies are not deleterious and serum treatment with dithiothreitol (DTT) should be performed to optimize crossmatch results [32].

New assays such as flow cytometry and the single antigen bead (SAB) assay, have emerged with increased sensitivity [33], although the clinical relevance of some of the anti-HLA antibodies detected could be doubtful. Flow cytometry crossmatching (FCXM) recognizes both complement-fixing and non-fixing anti-HLA antibodies using a polyclonal anti-human IgG conjugated with fluorochrome. This approach detects IgG1, IgG2, IgG3, and IgG4 isotypes of IgG and predicts the risk of graft failure in retransplanted patients with negative CDC [34]. This fact has been demonstrated in kidney transplantation in deceased [35] and living donors [36]. However, recipients with false-positive FCXM results (without the detection of DSA by other assays) exhibited comparable graft survival rates [37].

SAB assays use a monoclonal antibody that recognizes polyclonal human IgG (IgG1, IgG2, IgG3, and IgG4 isotypes) with increased sensitivity [33]. In those patients with negative CDC and FCXM results, the detection of DSA by SAB pre-transplantation is associated with an increased risk of cellular acute rejection and/or ABMR compared with those recipients without DSA. However, half of the patients with preformed DSA prior to transplantation, despite negative CDC and FCXM results, did not experience either cellular or antibody-mediated rejection [38].

Anti-HLA antibody detection based on SAB is now widely used in histocompatibility laboratories worldwide. However, it is important to know both the advantages and disadvantages of this test [39]. This is a key point for understanding the limitations of the SAB assay. The new tools for anti-HLA detection have improved the sensitivity of the assay but now the challenge is to identify the clinical deleterious anti-HLA antibodies (Table 1).

### 3.1. Caveats and Pitfalls in SAB Assays for Anti-HLA Antibody Detection

To better interpret SAB assay results, there are several points to keep in mind. One key factor to be considered is the semiquantitative measurement of the mean fluorescence intensity (MFI) performed using the Luminex instrument [40]. Without harmonization, the level of antibodies cannot be estimated accurately [41], leading to non-comparable results between different laboratories. The MFI value serves as an orientation tool, and with sera dilution, the prozone effect can be detected. By comparing net serum MFI values, variations in anti-HLA reactions with differing levels can be identified [42]. Therefore, each laboratory must set its own MFI threshold.

#### 3.1.1. False-Positive Results

Another important consideration is the bead panel design. For the screening of anti-HLA antibodies, the beads are primarily manufactured using natural HLA molecules. However, in SAB assays, the sources utilized by vendors differ, incorporating either recombinant or natural HLA panels [43] both of which are certified for qualitative interpretation. Additionally, non-specific reactions against denatured HLA molecules, cryptic antigens, and free beta2-microglobulin [44] can lead to false positive (FP) results. The density of HLA on the beads may be overrepresented compared to its natural expression on cells, particularly for the C, DQ, and DP loci [45]. The incidence of FP results is influenced by the cut-off value established by individual laboratories, with some studies reporting FP rates as high as 78% when using a cut-off of 1000 MFI [46]. To address false reactions, certain strategies have been proposed, such as comparing allele frequencies [47], using confirmatory tests from alternative vendors [48] or optimizing assay protocols (see next Section 3.1.2). Although the detection of de novo DSAs should be interpreted in the clinical context, FP results could mask other causes of graft function impairment.

#### 3.1.2. False-Negative Results

Low median fluorescence intensity (MFI) values should be interpreted with caution. Given the high sensitivity of the assay, low-titer anti-HLA antibodies may still be detected. These low levels could be attributed to HLA sensitization that occurred years prior, potentially due to events such as previous pregnancies, abortions, or transfusions (Figure 1). In such cases, HLAs may have elicited a transient immune response, characterized by rapid and short-term antibody production. It is important to recognize that even antibodies detected at subthreshold levels may still carry a potential risk of rebound upon re-exposure to the same HLA. Similar results could be obtained with some specificities against the same common reactive epitope group (CREG) or public epitope (Bw4/Bw6) [49]. One factor to consider is the “dilution” effect of the MFI signal [50] when anti-HLA antibodies against epitopes are present on different beads. However, this effect does not lead to a significant reduction in the MFI signal in several eplet-specific reactions [51]. Another potential cause of false-negative (FN) results is complement competition, which has been described as a key contributor to the prozone effect in anti-HLA testing by SAB [42]. To manage all these situations, the STAR group has defined a series of recommendations for the interpretation of the SAB test results [39]. The key points for anti-HLA antibody assessment are as follows: (a) Highly recommended: laboratories should implement measures to remove inhibition in the SAB test. Examples include the use of EDTA-treated sera, sera dilution studies, DTT treatment, or the heat inactivation of sera. (b) Moderate suggestions regarding MFI cut off value, between 1000 and 1500 may be considered for multi-center clinical trials. Additionally, in anti-HLA monitoring, differences of 25% in MFI values should not be considered clinically meaningful. All these approaches could be potentially adopted in histocompatibility laboratories worldwide.

A bead-by-bead normalization strategy has been recently published for the detection of weak anti-HLA antibodies [52]. The clinical impact of an FN result would potentially delay the diagnosis of AMR and treatment with an increased risk of graft loss [53].

### 3.2. Advantages of Anti-HLA Testing by SAB

One of the primary advantages of anti-HLA testing is its capability to determine reactions at high resolution (HR) at the two-field level, identify reaction patterns against cross-reactive groups (CREGs) and eplets, and predict potential reactions against HLA molecules that are not represented in the bead panel.

This option opened up an opportunity to change the way we assess the HLA MM for HLA molecular MM. However, to define the molecular MM between donor–recipient pairs, HR HLA typing of recipient and donors should be available. Currently, for deceased donors, there is no quick HR HLA typing test available. To overcome this problem, several web-based tools for HR HLA imputation are available, including Haplostats [54], HLA-EMMA [55], and Easy-HLA [56]. Nevertheless, imputed HR HLA typing has several pitfalls and is particularly skewed in non-Caucasian individuals [57]. Recently, a multiple-imputation approach has helped reduce errors in determining molecular MM [58].

Molecular MM represents a promising approach to enhance donor–recipient compatibility assessments and may become more relevant in the future when high-resolution HLA typing can be performed efficiently for deceased donors. Currently, several tools exist for estimating molecular MM. The predicted indirectly recognizable HLA epitope (PIRCHE) algorithm [59] estimates the disparity in T cells, while for B cells, eplet disparity (HLA matchmaker) [60] as well as solvent-accessible amino acid mismatch (HLA-EMMA) [55] disparity with comparable results [61] have been described. Other approaches, such as differences in amino acid number, hydrophobicity, and electrostatic charge have been proposed [62]. In addition to high-resolution typing, there are other hurdles to the consolidation of molecular MM as a tool to define the immunological risk between donor and recipient, including the lack of consensus on how to perform the calculations. Wiebe C. et al. described the independent role of class II eplet mismatch in dnDSA development [63] using a cut-off of >11 HLA-DR or -DQ eplet mismatch for the high-risk group. However, in a further study, the same group found single-molecule eplet mismatch to better discriminate against dnDSA development [64]. Davis et al. confirmed this approach in a UK cohort [65]. Evidence from pediatric heart transplantation points to the combined use of T and B molecular MMs to stratify the immunological risk better [66].

In addition to the requirement of having high-resolution donor and recipient HLA matching, there are several unresolved questions. For example, in the case of the HLA matchmaker, published studies are heterogeneous in combining antibody-verified and non-verified eplet MM. Moreover, the concept of eplet refers to a structural location within the HLA molecule, but some of them have not been shown to induce specific antibody-mediated reactions [67,68].

Along with all the issues described, it should also be noted that there are no large-scale validation studies of using the algorithms to calculate molecular MM, or extensive longitudinal studies to confirm their usefulness and ensure their applicability [69]. The integration of MM analysis into standard clinical workflows could potentially reduce graft immunogenicity, rejection risk, graft failure rates, patient sensitization, and retransplantation rates. However, further evaluation is needed [70]. The results of a public survey of the Canadian public on the implementation of epitope matching have been published, discussing several ethical issues [71]. This survey also provides the viewpoint of Canadian kidney transplant professionals who consider MM to be a promising technology to integrate into their allocation system once the ethical issues are resolved [72].

Another advantage of the SAB assay for anti-HLA is the enhanced characterization of anti-HLA reactions in patients on the waiting list. Furthermore, it has become possible to estimate the calculated panel reactive of antibodies (cPRA) in silico by comparing the reactions of anti-HLA antibodies (both complement-fixing and non-complement-fixing) with donor HLA-typing panels. Various cPRA calculators have been developed, including those from UNOS [73], the Canadian program [74], and Eurotransplant [75], as well as the Spanish PATHI program, all yielding comparable results [76]. The cPRA is the parameter used as a cut-off point in transplant access programs for highly sensitized (HS) patients. This approach to calculating sensitization has replaced PRA tests carried out in the traditional way in which the sera from patients on the waiting list were crossed with cells from donors by the CDC. The frequency of positive reactions was the PRA, so this value was adjusted to the possibility of having a positive CDC crossmatch reaction while the cPRA adjusts for the possibility of having a positive FCXM reaction.

Recently, the EuropeaN Guidelines for the mAnagement of Graft rEcipients (ENGAGE group) from the ESOT published a series of recommendations to estimate the immunological humoral risk between a patient and potential donor based on CDC, FCXM, virtual XM, and current and historic DSA [77,78]. The lowest-risk group comprises patients on the waiting list without detectable anti-HLA antibodies. The next level of immunological risk could be elicited by previous allorecognition due to transfusion, pregnancy, or a previous transplant involving re-exposure to the same HLAs, where historical DSA may have been detected but current results are negative. Patients with actual detectable DSA but with both CDC- and FCXM-negative results would be included in the intermediate humoral risk group.

The humoral risk increases when an actual positive DSA renders a positive FCXM with negative CDC, indicating a very high risk of AMR and accelerated chronic AMR. The highest humoral risk is observed in patients with DSA that evoke both FCXM- and CDC-positive results. In those cases, the transplantation is not allowed and would require desensitization protocols before transplantation.

With these new tools and the different types of anti-HLA defined, it is necessary to clarify which anti-HLAs are harmful and which are not. In organ exchange programs for HS patients, the transplant rate in patients who have a cPRA of 100% (cPRA-100) is significantly lower [79,80,81]. Therefore, new approaches are needed to provide viable options for cPRA-100 patients on the waiting list.

## 4. Delisting Strategies

Dilution serum studies have demonstrated that cPRA-100 patients are heterogeneous, and the reduction in cPRA after serum dilution could reflect the success of desensitization strategies before transplantation [82]. Moreover, the use of immune system evasion mechanisms, such as Streptococcus pyogenes-derived endopeptidase Imlifidase (IdEs) which targets human IgG [83], opens a window of opportunity for transplantation in cPRA-100 patients [84]. Clinical trials have demonstrated that IdEs are safe, immunogenic, and effective in cleaving DSA in patients with end-stage renal disease [85] with the ability to convert a positive crossmatch to a negative result within hours, enabling transplantation in patients who would otherwise be ineligible [86]. However, more recent clinical trials have not shown clear clinical benefits of the treatment of ABMR with IdEs compared with plasma exchange [87]. The first results of the combined experience of two independent open-label trials with 25 HS patients who underwent kidney transplantation were published in 2017 [88]. After 3 years of follow-up, the results showed comparable outcomes with other series of HS transplanted patients in terms of graft and patient survival with an increased rate of AMR [84]. Recently, a five-year follow-up of 39 HS patients treated with IdEs revealed an ABMR rate of 38% within the first 6 months of kidney transplantation, with no additional occurrences between 3 and 5 years ([89]. Another multicenter French study involving nine HS patients treated with IdEs with positive crossmatch showed an acceptable short-term efficacy in selected cases [90].

The utility of IdEs and daratumumab for desensitization in an HS patient with systemic lupus erythematosus and antiphospholipid syndrome for a blood-group incompatible living donor was demonstrated [91]. The first use of IdEs in a patient waiting for lung transplantation has also been reported [92]. However, concerns remain regarding the rebound to anti-HLA after transplantation [93,94]. Despite these preliminary data, further evidence from larger series is needed to confirm these results.

Meanwhile, other strategies for delisting in cPRA-100 patients with a lower immunological risk have been proposed [93]. The delisting strategy involves accepting HLA MM as a potential donor with anti-HLA current and/or historical positive reactions, facilitating access to transplantation. This approach assumes the risk of being transplanted with preformed DSA. Collecting CDC and FC crossmatch results will mark subsequent steps, which may conclude with treatment using IdEs [93]).

However, alternative strategies could be proposed to assess the immunological risk in cPRA-100 patients before using IdEs. Approaches based on MFI levels or shared epitopes have been proposed, but currently, there is no clinical evidence that validates these strategies. Furthermore, there is no consensus regarding the assignment of HLA for delisting. The French consensus guidelines for delisting in HS patients highlights HLA specificities with MFI levels below 5000 after serum dilution 1/10, and subsequently, if a positive crossmatch result follows, IdEs should be used [93]. The crucial role that histocompatibility laboratories play in assigning HLA specificities has recently been reviewed to decide if a transplant should be permitted [95], with interactions between the histocompatibility laboratory and clinical transplant teams being necessary.

Regarding the risks and benefits of delisting protocols that incorporate preformed DSA, European guidelines [77] recently compared different allocation systems and stressed that no European country has published a national consensus on the optimal management pathway for HS patients. Despite receiving priority in most of the donor-sharing programs, cPRA-100 patients remain on the waiting list for prolonged periods without a chance to receive a transplant and require a transplant across the HLA barrier with an increased rate of complications but also with increased benefits in terms of survival compared with those remaining on the waiting list [96]. One of the main complications after HS transplantation is ABMR [97]. In addition to conventional therapies used in transplant patients, HS patients face a high immunological risk and induction therapy with thymoglobulin or alemtuzumab is considered. In patients treated with IdEs, the start of induction therapy should be delayed, because endopeptidase also cleave rabbit IgG [98].

To avoid humoral injury to the graft, several treatments to remove HLA antibodies have been successfully implemented in clinical practice. These therapies include the removal of antibodies after plasmapheresis or immunoadsorption treatment [99], the use of IVIG as an immunomodulatory agent [100], the depletion of plasma and B cells with rituximab or ofatumumab (anti-CD20) [101], the inhibition of the complement cascade by use of eculizumab (anti-complement C5 factor) [102], and the suppression of the T cell-dependent antibody response [103].

Before IdEs treatment for desensitization, a short series of incompatible HLA (iHLA) kidney transplants demonstrated long-term results comparable to those of low-risk HS patients [104]. More recently, new evidence of successful iHLA kidney transplants after low-risk delisting strategies, reducing waiting list periods [105], and larger cohorts with low ABMR incidence and optimal graft function were described [106].

## 5. Discussion

SAB assays have allowed for a better characterization of anti-HLA antibody reactions after allosensitization. However, this has also led to an overestimation of forbidden anti-HLA reactions in patients on the waiting list. The better characterization of anti-HLA reactions, the history of previous HLA sensitization, the quantity of anti-HLA antibodies, and their ability to fix complement or not, have allowed the development of immunological risk scales to consider when carrying out delisting strategies with the aim of cPRA-100 patients being transplanted.

Despite the development of desensitization protocols, patients with cPRA-100 remain on the waiting list for longer periods even without donor offers. Although new treatments for HLA-incompatible transplantation with positive crossmatch are showing promising results, a better understanding of anti-HLA reactions would allow new strategies to address the scale of the immunological risk from different delisting strategies to create new chances for cPRA-100 patients to receive transplantation.

## Figures and Tables

**Table 1:** Description of assays used to detect anti-HLA antibodies.

Assay	Method	Anti-HLA Immunoglobulin Detected	Calculation of Sensitization Level
CDC	Complement-dependent cytotoxicity	IgG1	PRA
		IgG3	
		IgM	
ELISA	Enzyme-linked immunosorbent assay	Poli-IgG	
SAB	Single antigen bead assay	Poli-IgG	cPRA
FCXM	Flow cytometry	Poli-IgG	

CDC: complement-dependent cytotoxicity; cPRA: calculated panel reactive of antibodies; ELISA: enzyme-linked assay; FCXM: flow cytometry crossmatch; PRA: panel reactive of antibodies; SAB: single antigen bead assay.
